# Increased risk of Alzheimer’s disease in patients with psoriasis: a nationwide population-based cohort study

**DOI:** 10.1038/s41598-020-63550-2

**Published:** 2020-04-15

**Authors:** Miri Kim, Hyo Eun Park, Si-Hyung Lee, Kyungdo Han, Ji Hyun Lee

**Affiliations:** 1 Department of Dermatology, Yeouido St. Mary’s Hospital, College of Medicine, The Catholic University of Korea, #10, 63-ro, Yeongdeungpo-gu, 07345 Seoul, Korea; 2 Department of Dermatology, Seoul National University Hospital, Seoul, Korea; 3 Department of Medical Statistics, College of Medicine, The Catholic University of Korea, #222 Banpo-daero, Seocho-gu, 06591 Seoul, Korea; 4 Department of Dermatology, Seoul St. Mary’s Hospital, College of Medicine, The Catholic University of Korea, Seoul, #222 Banpo-daero, Seocho-gu, 06591 Seoul, Korea

**Keywords:** Dementia, Skin diseases, Alzheimer's disease, Risk factors

## Abstract

Although the pathogenesis of Alzheimer’s disease (AD) is unclear, neuroinflammation appears to play a role in its development. Psoriasis is a chronic inflammatory skin disease that has recently been found to genetically overlap with AD. We aimed to investigate the risk of AD in patients with psoriasis. Subjects with psoriasis (n = 535,927) and age- and sex-matched controls without psoriasis (at a 5:1 ratio; n = 2,679,635) who underwent ≥3 health examinations between 2008 and 2014 were included, drawn from the Korean National Health Insurance System database. There were 50,209 cases of AD (1.87%) in controls without psoriasis and 11,311 cases (2.11%) in patients with psoriasis, and the median follow-up was 3.35 years. In a multivariable-adjusted model, patients with psoriasis showed a significantly increased risk of AD (hazard ratio, 1.09; 95% CI, 1.07–1.12, p < 0.0001) compared to controls without psoriasis. Among patients with psoriasis, the risk of AD was significantly increased in psoriasis patients not receiving systemic therapy compared to those receiving systemic therapy (hazard ratio, 1.10; 95% CI, 1.08–1.12 *vs*. hazard ratio, 0.99; 95% CI: 0.90–1.09, p < 0.0001). The incidence of AD was significantly increased in patients with psoriasis compared to control subjects without psoriasis. Of note, systemic treatment for psoriasis was associated with a reduced risk of AD.

## Introduction

Alzheimer’s disease (AD), the most common cause of dementia in the elderly, is a chronic neurodegenerative disease affecting approximately 30 million people worldwide in 2015^1,2^. Although the pathogenesis of AD remains unclear, it is thought that genetic susceptibility, brain inflammation, and various environmental stimuli interact with each other to cause the disease. Recently, increasing evidence has implied that aberrant immune responses, particularly involving T-helper (Th)1/Th17 cells and cytokines, are involved in the inflammation associated with neurodegeneration in AD^3–5^.

Psoriasis is a Th1/Th17-mediated chronic inflammatory skin disease, and often develops between the ages of 15 and 30 years^6^. Genome-wide association studies (GWASs) have demonstrated genetic overlap between AD and psoriasis, and implied that inflammation could influence the pathogenesis and progression of AD^7^. Additionally, recent studies have suggested that psoriasis is associated with an increased risk of mental health disorders^8^ and Parkinson’s disease^6^. Since psoriasis occur much younger than AD, it seems plausible the effects of psoriasis could influence AD. These findings raise an important question concerning whether psoriasis and AD are linked. Previously, several studies have reported an association between AD and psoriasis, however, these were restricted to specific populations and included small sample sizes, or only assessed certain outcomes^9,10^.

Therefore, we conducted a population-based case–control cohort study using the Korean National Health Insurance System (NHIS) database to determine whether the incidence of AD is increased in patients with psoriasis.

## Results

### Baseline demographics of the study population

Baseline characteristics of participants are described in Table 1. Among the 535,927 patients with psoriasis, 499,488 (93.2%) patients (no systemic therapy group) had no evidence of receiving systemic agents for psoriasis, while 36,439 (6.8%) patients (systemic therapy group) had received systemic treatment for psoriasis.

**Table 1:** Baseline demographics of patients with psoriasis and controls without psoriasis.

	Control	Psoriasis group (n = 535,927)		P value
		No systemic therapy group	Systemic therapy group	
**Total number (n)**	2,679,635	499,488	36,439	<0.0001
**Age 40–64 years (%)**	1,915,435 (71.48)	354,129 (70.9)	28,958 (79.47)	<0.0001
**Male (%)**	1,436,325 (53.6)	266,333 (53.32)	20,932(57.44)	<0.0001
**Mean age (years)**	57.55 ± 11.7	57.71 ± 11.76	55.34 ± 10.6	<0.0001
**Mean follow-up duration (years)**	3.35 ± 2.01	3.36 ± 2.01	3.13 ± 2.03	<0.0001
**Income lowest quintile (%)**	651,672 (24.32)	130,109 (26.05)	9,277 (25.46)	<0.0001
**Comorbidity (%)**				
Diabetes mellitus	281,542(10.51)	67,741 (13.56)	4,742 (13.01)	<0.0001
Hypertension	741,485 (27.67)	160,673 (32.17)	10,008 (27.47)	<0.0001
Dyslipidemia	413,393 (15.43)	99,328 (19.89)	6,808 (18.68)	<0.0001

### Risk of AD in patients with psoriasis compared to controls without psoriasis

After a mean follow-up period of 3.35 ± 2.01 years, the incidence of AD was 5.6 per 1,000 person-years in controls without psoriasis and 6.3 per 1,000 person-years in patients with psoriasis (no systemic therapy group: 6.5 per 1,000 person-years; systemic therapy group: 3.7 per 1,000 person-years, p for trend < 0.0001) (Table 2). The mean duration between the diagnosis of psoriasis and AD after psoriasis was 2.45 ± 1.67 years in controls and 2.42 ± 1.67 years in patients with psoriasis (Table 2). The duration between psoriasis and AD diagnosis actually reflect the time between psoriasis and AD diagnosis. In the control group, the control subject was followed up from the first diagnosis of psoriasis in psoriasis patient matched with each control subject.

**Table 2:** Hazard ratios and 95% confidence intervals of Alzheimer’s disease according to the presence of systemic therapy for psoriasis.

Group	Event (n)	Duration*, years (mean ± SD)	Incidence rate (per 1000 person-years)	Model 2	Model 3
**Control**	50,209	2.45 ± 1.67	5.586	1 (Ref.)	1 (Ref.)
**Psoriasis group**	11,311	2.42 ± 1.67	6.306	1.133 (1.11, 1.156)	1.093 (1.071, 1.116)
No systemic therapy group	10,889		6.483	1.137 (1.114, 1.161)	1.098 (1.075, 1.121)
Systemic therapy group	422		3.698	1.023 (0.928, 1.124)	0.988 (0.896, 1.086)

^*^Mean duration between the incident psoriasis and AD diagnosis.

Model 2: adjusted by age and sex.

Model 3: adjusted by age, sex, income level, diabetes mellitus, hypertension, dyslipidemia and depression.

**Figure 1:**
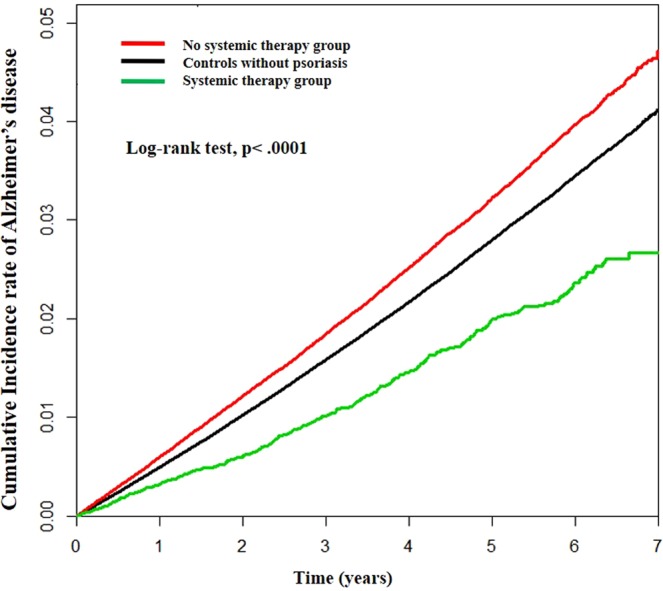
Alzheimer’s disease (AD) in patients with psoriasis. The estimated cumulative incidence of AD is significantly higher in psoriasis patients than in control subjects without psoriasis. In addition, psoriasis patients who received systemic therapy showed a lower incidence of AD than psoriasis patients without any systemic therapy for psoriasis by Kaplan-Meier analysis. (Log-rank test, p < 0.0001).

As illustrated in a Kaplan–Meier plot, subjects with psoriasis showed a greater tendency to suffer from AD compared to controls without psoriasis. However, the risk of AD was decreased in the systemic therapy group compared to controls without psoriasis (log-rank test; p < 0.0001, Fig. 1). After adjusting for age, sex, income level, diabetes, hypertension, dyslipidemia and depression (model 3), patients with psoriasis showed a significantly increased risk of developing AD (HR, 1.09, 95% CI: 1.07–1.12, p < 0.0001) compared to controls without psoriasis (Table 2).

### Subgroup analyses

**Figure 2:**
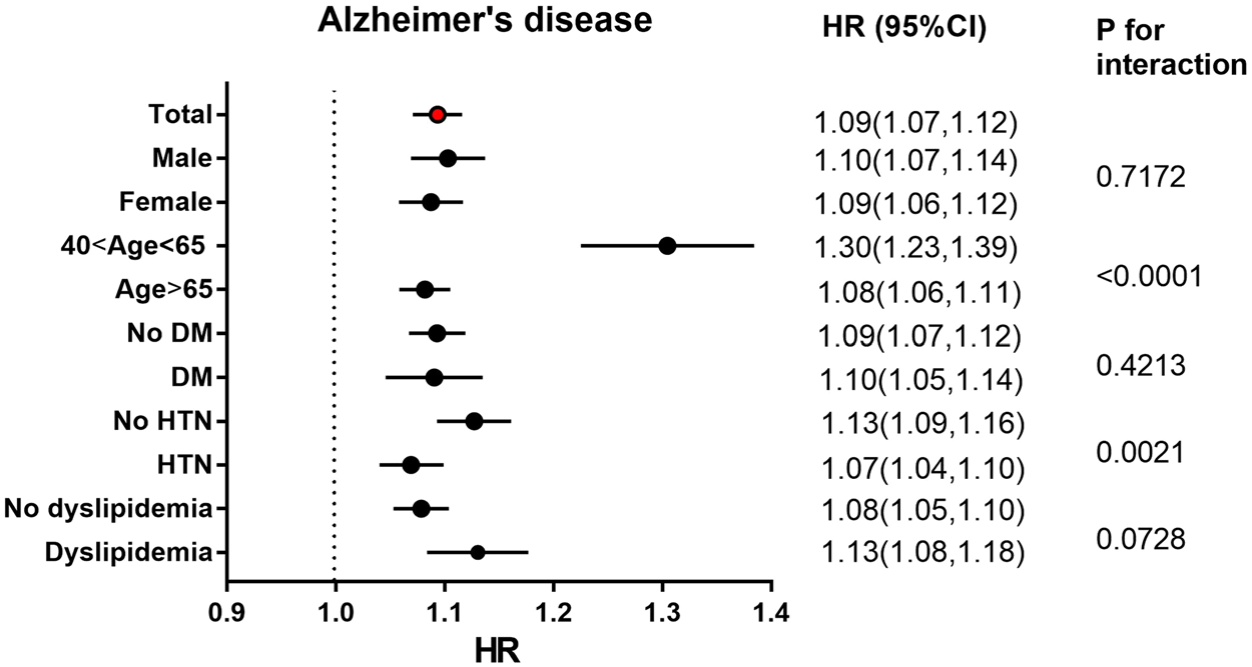
Hazard ratios and 95% confidence intervals of Alzheimer’s disease in psoriasis group vs. controls without psoriasis in subgroups. Adjusted for age, sex, income level, diabetes mellitus (DM), hypertension (HTN), dyslipidemia, and depression.

An analysis stratified by age, sex, and presence or absence of diabetes, hypertension, or dyslipidemia was conducted. Higher adjusted HRs for developing AD were observed among males, and among those without diabetes or hypertension, and in those with dyslipidemia; there were no significant differences in analyses comparing incidence rates between the sexes, diabetes, or dyslipidemia status (Table 3). However, there was a significantly higher incidence of AD in individuals without hypertension (HR = 1.13 vs 1.07, p-value = 0.0021) (Fig. 2). Of note, younger patients (40–64 years) with psoriasis had a significantly higher incidence of AD than that of older patients (≥65 years) (HR, 1.30, 95% CI: 1.23–1.39 *vs*. HR, 1.08, 95% CI: 1.06–1.11, *p* for interaction <0.0001) (Fig. 2).

**Table 3:** Subgroup analyses of the risk of Alzheimer’s disease among control, psoriasis patients treated with or without systemic therapy according to sex, age, presence or absence of diabetes mellitus, hypertension and dyslipidemia.

Subgroup		Event(n)	Incidence rate (per 1000 person-years)	HR (95%CI)	
				Model 3	P for interaction
Male	**Control**	21,680	4.472	1 (Ref.)	0.1987
	**Psoriasis group**	4,913	5.077	1.103 (1.069, 1.137)	
	No systemic therapy	4,718	5.234	1.112 (1.077, 1.147)	
	Systemic therapy	195	2.939	0.922 (0.798, 1.058)	
Female	**Control**	28,529	6.889	1 (Ref.)	
	**Psoriasis group**	6,398	7.747	1.087 (1.058, 1.117)	
	No systemic therapy	6,171	7.931	1.088 (1.059, 1.119)	
	Systemic therapy	227	4.753	1.056 (0.924, 1.2)	
40 y < Age < 65 y	**Control**	4,852	0.743	1 (Ref.)	<0.0001
	**Psoriasis group**	1,333	1.022	1.303 (1.226, 1.385)	
	No systemic therapy	1,265	1.044	1.32 (1.24, 1.404)	
	Systemic therapy	68	0.739	1.056 (0.823, 1.33)	
Age > 65 y	**Control**	45,357	18.430	1 (Ref.)	
	**Psoriasis group**	9,978	20.386	1.082 (1.058, 1.105)	
	No systemic therapy	9,624	20.592	1.085 (1.062, 1.109)	
	Systemic therapy	354	16.038	0.99 (0.89, 1.097)	
No DM	**Control**	39,661	4.899	1 (Ref.)	0.8578
	**Psoriasis group**	8,386	5.377	1.093 (1.067, 1.119)	
	No systemic therapy	8,082	5.538	1.097 (1.071, 1.124)	
	Systemic therapy	304	3.035	0.983 (0.877, 1.099)	
DM	**Control**	10,548	11.794	1 (Ref.)	
	**Psoriasis group**	2,925	12.491	1.09 (1.046, 1.135)	
	No systemic therapy	2,807	12.746	1.094 (1.05, 1.141)	
	Systemic therapy	118	8.462	0.994 (0.824, 1.185)	
No HTN	**Control**	24,495	3.716	1 (Ref.)	0.0043
	**Psoriasis group**	4,989	4.041	1.127 (1.093, 1.161)	
	No systemic therapy	4,790	4.162	1.133 (1.099, 1.169)	
	Systemic therapy	199	2.380	0.982 (0.852, 1.126)	
HTN	**Control**	25,714	10.724	1 (Ref.)	
	**Psoriasis group**	6,322	11.307	1.069 (1.04, 1.099)	
	No systemic therapy	6,099	11.538	1.073 (1.043, 1.103)	
	Systemic therapy	223	7.310	0.984 (0.86, 1.119)	
No dyslipidemia	**Control**	39,940	5.150	1 (Ref.)	0.1967
	**Psoriasis group**	8,340	5.669	1.078 (1.053, 1.104)	
	No systemic therapy	8,031	5.836	1.083 (1.057, 1.109)	
	Systemic therapy	309	3.254	0.976 (0.871, 1.09)	
Dyslipidemia	**Control**	10,269	8.325	1 (Ref.)	
	**Psoriasis group**	2,971	9.213	1.13 (1.084, 1.177)	
	No systemic therapy	2,858	9.422	1.135 (1.088, 1.183)	
	Systemic therapy	113	5.897	1.02 (0.843, 1.222)	

Abbreviations: CI, confidence interval; HR, hazard ratio; DM, diabetes mellitus; HTN, hypertension.

Model 3: adjusted by age, sex, income level, diabetes mellitus, hypertension, dyslipidemia and depression.

## Discussion

In this nationwide study, we found a significantly increased risk of newly diagnosed AD among patients with psoriasis compared to age- and sex-matched controls without psoriasis. This association was significantly stronger in middle-aged patients than in elderly patients (≥65 years) with psoriasis (HR: 1.30 *vs*. HR: 1.08). We also observed that those patients with psoriasis who were treated with systemic therapy had a lower risk of AD than that of controls without psoriasis.

Although the exact mechanism of AD has not been fully elucidated, increasing evidence has implied that neuroinflammation plays an important role in its development^11–13^. In AD, the activation of microglial cells, the key inflammatory cells in the brain, induces the release of proinflammatory mediators, resulting in neuronal damage^14^. In addition, IL-12/IL-23 signaling has been implicated in the development of amyloid-induced neurodegeneration^4^. Indeed, blocking the common p40 subunit of IL-12 and IL-23 reduced the number of amyloid β plaques, an important pathology of AD, and appeared to improve the cognitive deficits in a mouse model of AD^3^. The IL-23/T helper 17 axis is considered to be the most important factor in the development of psoriasis, and anti-IL-12/23 p40 monoclonal antibody, a drug targeting this axis, is used worldwide as a treatment of psoriasis^15^. Furthermore, GWASs have revealed a genetic overlap between AD and psoriasis, suggesting that an immunological mechanism plays a role in the pathogenesis of AD^7,16,17^. In a cross-sectional pilot study that assessed 41 patients with psoriasis and 37 controls using neuropsychological tests, Gisondi *et al*. reported that the incidence of mild cognitive impairment was higher in patients with chronic plaque psoriasis than in the controls, implying that patients with psoriasis are at an increased risk of developing AD^10^. In line with these overlapping inflammatory pathways and shared genetic risk loci, we observed that patients with psoriasis have a higher risk of AD compared to the general population using the NHIS database.

Notably, we observed a significant reduction in the incidence of AD among patients with psoriasis prescribed systemic medications (acitretin, methotrexate, cyclosporine, and biologic agents) compared to controls without psoriasis. The pathophysiological concept of CVD is widely accepted as a chronic inflammatory condition, and epidemiologic studies have shown that the systemic anti-inflammatory drugs such as biological drugs and methotrexate can attenuate the risk of CVD in patients with severe psoriasis compared to other anti-psoriatic treatments^18–24^. Recently, Lee *et al*. reported that a significantly increased risk of Parkinson’s disease was observed in the systemic therapy exposed-psoriasis group compared to the unexposed-psoriasis group^6^. In addition, it has been reported that acitretin, which is frequently used as a treatment for psoriasis in Korea^25^, enhances the gene expression of the metalloproteinase ADAM 10, thereby inhibiting Aβ peptide production and preventing the formation of amyloid plaques in a mouse model of AD^26,27^. Several clinical trials to examine the effect of TNF inhibitors in patients with AD have demonstrated their considerable beneficial effects on cognitive and behavioral improvement^28,29^. In this sense, we assume that systemic anti-inflammatory drugs reduce the inflammation of neuronal cells, lowering overall inflammation in the body, which has a preventive effect on AD.

In this study, age-stratified analyses (age and sex matched) revealed that the association between psoriasis and AD was more pronounced in middle-aged (40–64 years) compared to elderly (≥65 years) patients (HR 1.30 *vs*. 1.08, p < 0.0001). Although the absolute number and incidence of events were high above 65 years and low under 65, individuals who develop psoriasis at younger ages (40–60 years) are more likely to develop AD than age-matched individuals who are not diagnosed with psoriasis. In this regard, previous studies have shown that aging promotes a decrease in T cell immune function thereby reducing inflammation and leads to suppress the development of skin inflammatory diseases, which may support our hypothesis^30–32^. Additionally, since elderly people have various chronic diseases such as diabetes mellitus, hypertension, hyperlipidemia and depression and take many kinds of drugs, the effect of psoriasis on the development of AD may be more sensitive in middle age than in older age^33,34^. However, our results were meaningful after adjusting for these confounding variables, implying that reduced T cell immunity due to aging and weakened disease activity in psoriasis itself leads to the weak association between psoriasis and AD.

There were several limitations to our study. First, the subjects were identified for study inclusion based only on registered diagnostic codes. Nevertheless, the diagnoses are relatively accurate, because a diagnosis is covered by the NHIS in Korea only after the diagnosis is confirmed by the Mini-Mental State Examination (MMSE). Second, the nationwide database lacks detailed data on the severity and type of psoriasis, as patients with psoriasis were only classified as having received a systemic treatment or not. Third, because our study was a retrospective epidemiological study, it was not possible determine a causal relationship accurately. To avoid the possible effects of reverse causality, we established a 12-month washout period and excluded subjects with pre-existing psoriasis or AD. Fourth, we did not have information that could affect the incidence of AD such as education status, smoking, alcohol consumption and family history of AD. Fifth, it is well known that pathogenic variants in genes such as the APP, PSEN1, and PSEN2 are associated with early onset familial AD (ref)^35^. However, since the patients’ genetic information is not included in this study, it is difficult to know whether the genetic predisposition related to early AD may have affected our findings that younger patients (age <65 years) with psoriasis are more prone to AD than older patients (≥65 years). Further research is needed to explore the relationship between psoriasis and early versus late onset AD. Lastly, since our research is based on nationwide data representative of the Korean population, it is difficult to generalize this result to other racial/ethnic populations. Despite these limitations, this is the large-scale, population-based cohort study to evaluate the association between AD and psoriasis. The NHIS database covers nearly 100% of the Korean population, and our study provides the first evidence that the use of systemic anti-inflammatory drugs can reduce the risk of AD.

In conclusion, we found a significantly increased risk of AD in Korean patients with psoriasis compared to a matched control group. These results indicate that chronic inflammatory conditions in psoriasis may have an important impact on the nervous system and thus in increasing the risk of developing AD. Further studies are needed to explore the potential causes of the increased risk of AD and whether these effects vary depending on the type of systemic treatment used in psoriasis.

## Methods

### Data sources

The NHIS database used in this study covers approximately 97.2% of the population of Korea^36^. This system reviews inpatient and outpatient claims sent from healthcare institutions. The database includes information on patient age, sex, diagnoses and comorbidities based on the International Classification of Disease, 10^th^ revision (ICD-10), and records of prescriptions, procedures, and prescribed drugs^37^. Enrollees in the NHIS are assigned unique identification codes, and are recommended to have a health checkup at least every 2 years^38^. This study was approved by the institutional review boards of the Catholic University of Korea (no. KC17ZESI0311) and Seoul St. Mary’s Hospital (No. KC17ZESI0505). The informed consent was waived because all data were anonymized and de-identified.

### Study population

From the NHIS database, we enrolled patients aged 40 year or older who of age who underwent health screenings from January 2008 to December 2014. Among these subjects, patients who were diagnosed at least once with psoriasis (ICD-10 codes: L40) at clinics or hospitals were selected from the database. We excluded subjects with missing data on at least one variable. To avoid confounding by pre-existing diseases, those who had a history of AD (ICD-10 codes: F00 or G30) before the index year were also excluded. Ultimately, the study population consisted of 535,927 subjects.

We analyzed subjects with psoriasis (n = 535,927) and controls without psoriasis (n = 2,679,635) who were randomly selected according to a 5:1 ratio with age- and sex-matched subjects during the same period. Patients with psoriasis were divided into two groups; systemic therapy group: patients who received systemic treatment such as acitretin, methotrexate, cyclosporine and biologics, no systemic therapy group: patients who were not treated with any systemic agent.

### Study outcomes

This study investigated newly diagnosed patients with AD. The definition of AD was based on the presence of relevant ICD-10 codes (F00 or G30) and the prescription of medication for AD (galantamine, rivastigmine, donepezil, or memantine), as determined using the NHIS claims database. When codes for AD both and other types of dementia were recorded, we followed the principal diagnosis. If both code types were included in additional diagnoses up to the second claim database, the subject was classified as having ‘other’ dementia. The patients diagnosed with AD prior to this study enrollment were excluded. This study was initiated after a 12-month washout period between 2006 and 2007 to reduce the confounding effect of previously diagnosed AD on the study outcome. The study population was followed from baseline to the date of incident AD or until December 31, 2014, whichever came first.

### Statistical analysis

Baseline demographic characteristics are presented as means ± standard deviation or numbers (%). The incidence of AD was calculated by dividing the total number of incident cases by the entire follow-up duration (person-years). The cumulative incidence of AD according to the presence of psoriasis and systemic therapy for psoriasis was calculated using Kaplan–Meier curves, and the log-rank test was used to analyze differences between the groups. Hazard ratios (HRs) and 95% confidence intervals (95% CIs) for the risk of AD were determined using Cox proportional hazards models after adjusting for multiple confounding variables. Model 2 was adjusted for age and sex, and model 3 was further adjusted for income level, diabetes, hypertension, dyslipidemia, and depression. We performed subsequent subgroup analyses and interaction testing using a likelihood ratio test to evaluate the difference in the risk of AD according to subgroups. All statistical analyses were performed using SAS ver. 9.4 (SAS Institute, Cary, NC, USA).

### Ethics approval and consent to participate

This study was approved by the institutional review boards of the Catholic University of Korea (no. KC17ZESI0311) and Seoul St. Mary’s Hospital (No. KC17ZESI0505). Informed consent was not obtained because anonymous and de-identified information was used for the analysis.
